# Assessment of upper GI motor activity and GI symptoms in patients with amyotrophic lateral sclerosis: an observational study

**DOI:** 10.3389/fneur.2024.1509917

**Published:** 2025-01-13

**Authors:** Emanuela Ribichini, Nadia Pallotta, Danilo Badiali, Maria Carlucci, Marco Ceccanti, Chiara Cambieri, Laura Libonati, Enrico Stefano Corazziari, Giovanni Ruoppolo, Maurizio Inghilleri

**Affiliations:** ^1^Department of Translational and Precision Medicine, Sapienza University, Rome, Italy; ^2^Neuromuscular Disorders Unit, Department of Human Neurosciences, Sapienza University, Rome, Italy; ^3^Department of Gastroenterology, Istituto Clinico Humanitas, Rozzano, Italy; ^4^Department of Sense Organs, Sapienza University, Rome, Italy; ^5^Department of Human Neurosciences, Sapienza University, Rome, Italy; ^6^Istituti di Ricovero e Cura a Carattere Scientifico (IRCCS) Neuromed, Pozzilli, IS, Italy

**Keywords:** amyotrophic lateral sclerosis, gastric emptying, esophageal manometry, esophageal motility, upper esophageal sphincter, autonomic nervous system, vagus nerve

## Abstract

**Background/aims:**

Oro-pharyngeal dysfunction has been reported in Amyotrophic Lateral Sclerosis (ALS). We aimed to assess ALS patients upper gastrointestinal (GI) motor activity and GI symptoms according to bulbar and spinal onset and severity of ALS.

**Methods:**

ALS bulbar (B) and spinal (S) patients with ALS Functional Rating Scale (ALSFRS-r) ≥35, bulbar sub-score ≥10, and Forced Vital Capacity (FVC) >50%, underwent to: Fiberoptic Endoscopic Evaluation of Swallowing (FEES); esophageal manometry; gastric emptying; Rome symptom questionnaire. Medical Research Council Scale for Muscle Strength (MRC) was performed for the upper and lower limbs. Mann-Whitney's U, Fisher's ranks test, Pearson's test was used.

**Results:**

Thirteen ALS patients were included (6 F; mean age 61.2 ± 13.7 years, range: 37-87), 5 with B and 8 with S onset (ALSFRS-R score 39.5 ± 4.9, MRC score 128.6 ± 23.3, disease duration 22.8 ± 17.9 months). FEES detected a high dysphagia score in 5 patients with no difference between S and B phenotype. Lower esophageal sphincter pressure was normal in all patients. Esophageal dysmotility was observed in three S and two B onset patients. Upper esophageal sphincter (UES) pressure was higher in all ALS patients. UES spasms and delayed gastric emptying were detected in two B and one S and in two B and four S patients, respectively. There was no correlation between esophagogastric motor abnormalities and clinical characteristics of ALS, nor GI symptoms.

**Conclusions:**

The presence of UES spasm and the delayed gastric emptying in a subgroup of ALS patients may suggest the role of ANS dysfunction in ALS.

## Introduction

Amyotrophic lateral sclerosis (ALS) (1) is a neurodegenerative disease causing progressive physical disabilities. The upper and lower motor neurons are primarily affected, but involvement of the sensory nervous system has also been reported (2). ALS has different courses, but progressive paralysis leads to death from respiratory failure within 2 or 3 years from the diagnosis in the case of bulbar, and 3–5 years in the case of spinal onset, respectively (1). Despite the selective involvement of motor neurons, the presence of non-motor symptoms and involvement of autonomic cardiac and sudomotor regulation have been recently demonstrated in the more advanced stages of the disease (3). About one-third of patients show subclinical cardiac, vascular, genitourinary, and gastrointestinal involvement (4, 5). Nevertheless, the evaluation of these symptoms is made difficult due to the poor prognosis of the disease and the small number of subjects analyzed in the studies.

Dysphagia is one of the most disabling gastrointestinal (GI) symptoms in ALS (6) and is due to failure of oral and pharyngeal swallowing associated with progressive involvement of brainstem motor neurons (4). A sensory deficit of the larynx unrelated to a motor deficit has been found in one-third of ALS patients (2, 7). As the disease progresses, swallowing becomes increasingly difficult in all patients, favoring malnutrition and aspiration pneumonia.

In ALS patients, combined video-fluoroscopic and manometric evaluation demonstrated progressive reduction of pressures first in the oropharynx and then in the hypopharynx associated with episodes of upper esophageal sphincter (UES) spasm (7). Several studies have reported oropharyngeal dysfunction in ALS patients, but little is known about changes in motor activity of the esophagus and stomach.

ALS patients also complain of constipation, abdominal pain, abdominal fullness, and nausea (4), which may be secondary to an involvement of the autonomic nervous system (ANS) (9). In the advanced stages of the disease, 80% of ALS patients present an alteration of gastric motor activity (10) but it is not known whether gastric emptying is delayed at the onset of ALS disease.

Therefore, this prospective observational pilot study aimed to evaluate in ALS patients, (a) the oro-pharyngeal phase of swallowing and esophageal motility using manometry and Fiberoptic Endoscopic Evaluation of Swallowing (FEES) and (b) gastric emptying by ultrasound (US).

A secondary aim was to evaluate the association of pharyngo-esophageal and gastric motor function with GI symptoms, according to bulbar and spinal onset, and the severity of ALS disease assessed with the functional ALSFRS-R scale.

## Methods

### Subjects

Consecutive ALS patients referring to the Center of Rare Neuromuscular Diseases, University of Rome “Sapienza,” were evaluated following written informed consent and the approval of the Local Ethical Committee (#2174/16.06.2016).

As inclusion criteria, patients were at an intermediate level of disease according to the ALS Functional Rating Scale (ALSFRS-r) (≥35), bulbar sub-score ≥10 (11), a Forced Vital Capacity (FVC) >50%, and able to take an oral meal. The Medical Research Council Scale for Muscle Strength (MRC) for upper and lower limbs was performed.

None of the patients had other motor neuron disease and/or systemic diseases with associated alterations of the ANS. None of the patients underwent tracheotomy, enteral, or parenteral nutrition. None had severe psychiatric disorders or had traumatic, neoplastic, cerebrovascular, or other neurodegenerative diseases requiring PEG.

Patients were interviewed with Rome III diagnostic criteria standardized questionnaire made of 50 items, inquiring on demography, daily habits, alcohol consumption, smoking, past medical history, gastroesophageal and intestinal symptoms, and bowel pattern.

### Clinical and fiberoptic endoscopic evaluation of swallowing of oropharyngeal phases of swallowing

The following variables were evaluated with the FEES examination: pharyngeal and laryngeal morphology, velopharyngeal, and laryngeal motility, sensitivity of the larynx, including the study of the laryngeal adductor reflex (LAR). Based on these parameters, the Penetration Aspiration Scale (PAS), the Pooling score (P-score), and the P-SCA score were fulfilled. The PAS evaluates the severity and depth of aspirations, according to a scale of 8 points: the patient is considered dysphagic when the score is above 1 (12). The P-score assesses the control capacity of bolus and residues (13) and a P score >5 is indicative of the risk of dysphagia; when it is integrated with some parameters such as age, collaboration, and pharyngeal sensitivity, the P-SCA score is obtained (14). Both scores of dysphagia are expressed as numerical values (3–4 = absent; 5–8 = mild; 9–12 = moderate; 13–16 = severe).

### Esophageal manometry

High-resolution manometry (HRM) or conventional perfusion manometry was performed after passing the catheter through the nose of the fasting patient lying in the supine position. At HRM after identification of UES, LES landmarks, and respiratory inversion point (RIP), esophageal motor activity was assessed during a series of ten swallows of 5 ml water boluses. At traditional manometry, intraluminal pressure was recorded by an eight-lumen manometric catheter with radially oriented side holes located 5 cm apart. The manometric probe was located with its recording part within the stomach and then withdrawn by a stationary pull-through technique during at least five swallows of 5-ml water boluses and finally positioned with its proximal side hole at the level of UES.

LES resting pressure (pLES) was determined with pull-through maneuvers during suspended respiration. Normal peristaltic activity was defined according to reference standards (15) as the presence of contraction waves of amplitude >40 mmHg that propagate ab oral without interruptions or DCI > 450 mmHg-cm-s. The normal range of LES resting pressure was between 10 and 40 mmHg (16). UES parameters were compared with those of a control group of 14 sex- and age-matched patients without symptoms such as globus or dysphagia and/or signs of pharyngo-esophageal motor abnormalities.

### Gastric antral volume and gastric emptying time

Gastric emptying was evaluated by measuring gastric antral volume with ultrasonography (4 MHz linear probe, Tosbee, Toshiba, Japan). After an overnight fast, the subjects ate an ordinary standard solid meal of 525 kcal (70 g of bread, 40 g of ham, 40 g of cheese, and 250 ml of water; 50% carbohydrates, 25% lipids, 25% proteins). The study was carried out with patients in orthostatism, in fasting condition, after the end of meal ingestion, and at 30-min intervals thereafter over a total period of 180 min. Between measurements, subjects could move freely. Delayed gastric emptying was defined as the final antral volume, at 180 min exceeding the mean value plus 2DSs compared with the 28 healthy volunteers according to a published study (17).

### Protocol of study

After the inclusion visit, patients were submitted in random order and on different days: (a) to clinical and fiberoptic endoscopic evaluation of oropharyngeal phases of swallowing (FEES); (b) esophageal manometry; (c) gastric emptying evaluation after caloric meal.

The operators (GR, DB, NP) were blind to the patient's phenotype.

### Statistical analysis

Continuous descriptive variables are expressed as mean median and standard deviation. Mann-Whitney's U test for ranks for continuous variables and Fisher's test for dichotomous variables were used for comparisons between groups. The relationship between two quantitative variables was assessed using Pearson's independence test. The level of significance was set to *p* < 0.05.

## Results

Thirteen ALS patients (6 women and 7 men; mean age 61.2 ± 13.7 years, range 37–87 years) met the inclusion criteria and were enrolled, eight patients with spinal and five with bulbar onset. The mean duration of the disease was 22.8 ± 17.9 months (median 10 months). The patients showed a mean ALSFRS-R score of 39.5 ± 4.9 (median 42) and a mean MRC score of 128.6 ± 23.3 (median 150). Demographic and clinical characteristics of ALS patients and spinal and bulbar onset are described in Table 1.

**Table 1:** Demographic and clinical characteristics of ALS patients and spinal and bulbar onset.

	**BULBAR (*n* = 5)**	**SPINAL (*n* = 8)**	**Z-score**	***P*-value**
Age at onset (years)	65.6 ± 12.6	55.5 ± 14.3	1.17	0.2
ΔD-Test (months)	9.2 ± 6.1	31.3 ± 17.6	2.19	0.02
ALSFRS-R	42.4 ± 4.7	37.7 ± 4.4	−1.68	0.09
MRC (global score)	146.6 ± 5.6	117.5 ± 23.3	−2.56	0.01

ΔD-test, interval between ALS diagnosis and pharyngo-esophageal-gastric evaluation test; ALSFRS-R, Amyotrophic Lateral Sclerosis Functional Rating Scale; MRC, Medical Research Council Scale for Muscle Strength.

### Esophageal motility

**Figure 1:**
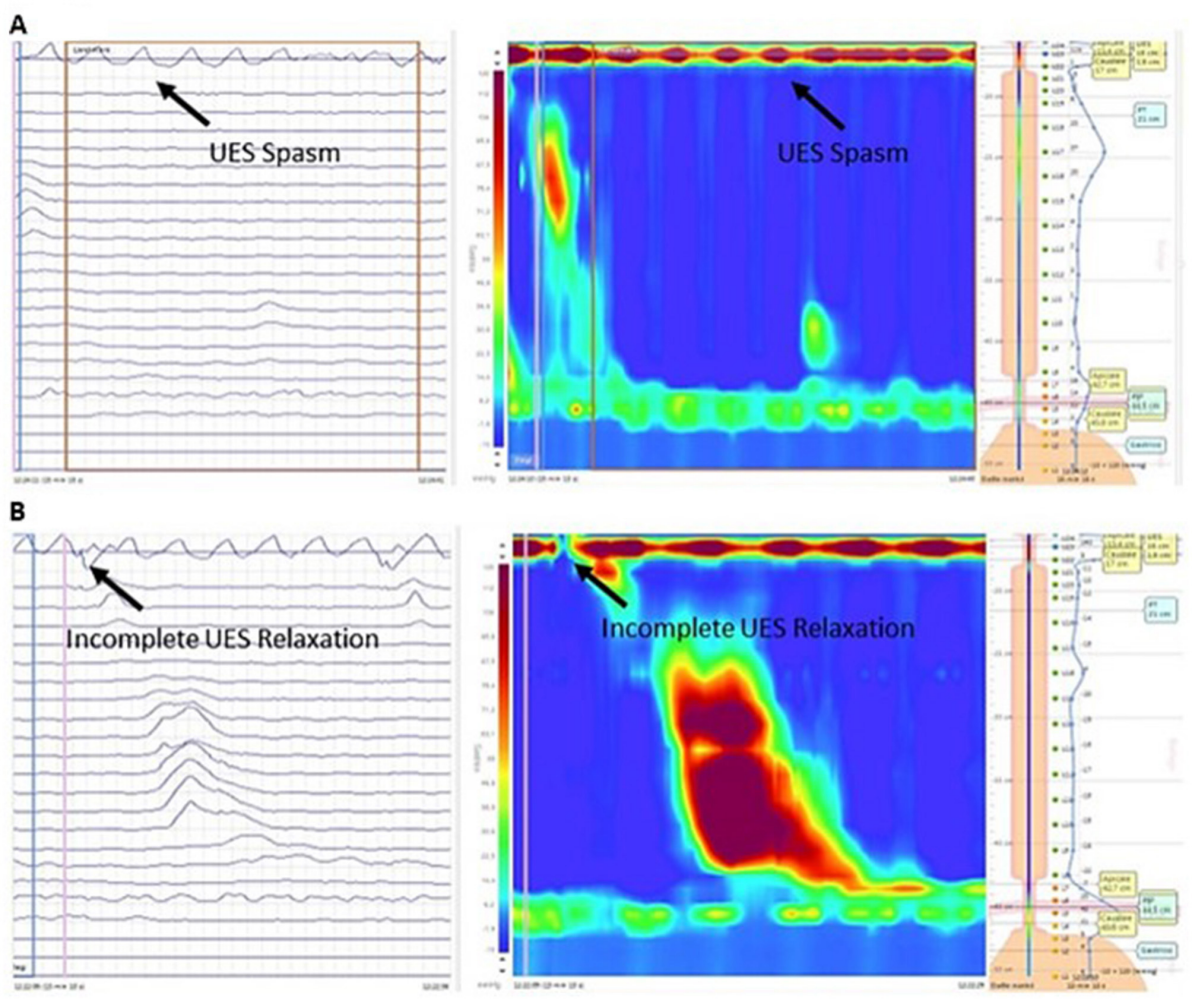
Esophageal HRM (left-side conventional line tracing and right-side pressure topography plot) in a bulbar ALS patient during a wet swallow of a 5-mL bolus of water. **(A)** Episodes of UES spasm. **(B)** Incomplete UES relaxation. HRM, High-Resolution Manometry; ALS, Amyotrophic lateral sclerosis; UES, Upper esophageal sphincter.

**Figure 2:**
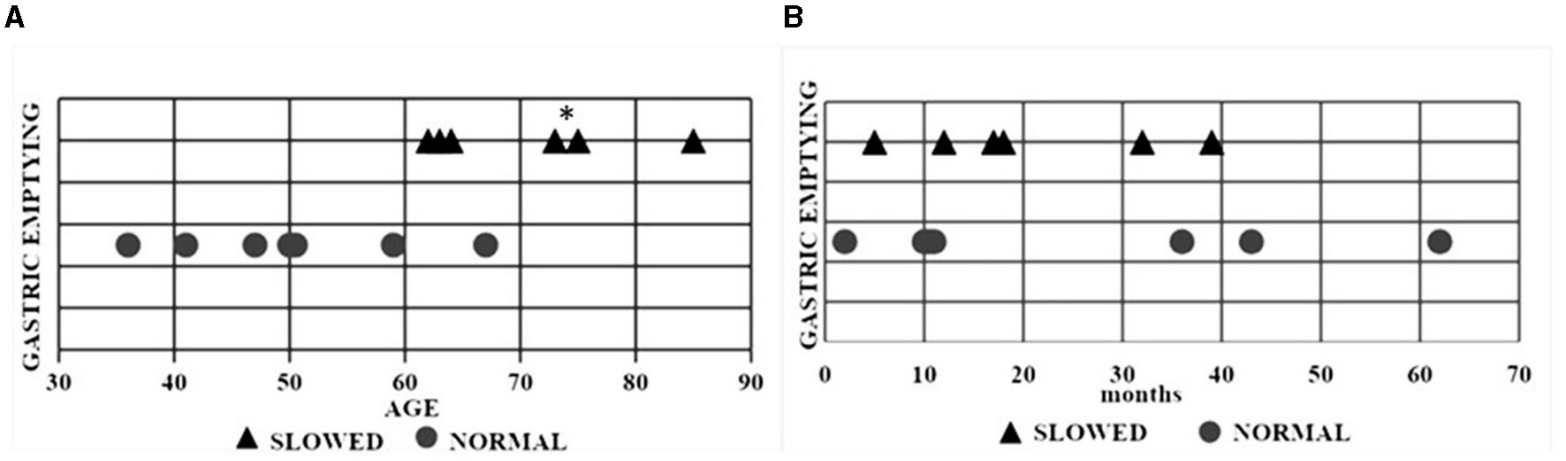
Scatter plot of each ALS patient with normal and delayed gastric emptying. **(A)** Relationship between gastric emptying and age. **(B)** Relationship between gastric emptying and disease duration. Triangular symbols indicate a slowed gastric emptying and round symbols a normal gastric emptying. *p = 0.012. ALS, Amyotrophic lateral sclerosis.

Twelve patients underwent an esophageal manometry, and one patient refused. HRM was performed in all control patients and seven ALS patients, and conventional perfusion manometry in the remaining five patients. There were no significant age and gender differences between ALS patients and controls (data don't show). The mean (and median) pUES was higher in ALS patients than in controls (67.8 ± 37.9 mmHg, median 55 mmHg vs. 37.1 ± 21.1 mmHg median 30.5 mmHg, *p* = 0.02), with no differences between the spinal (58 ± 43 mmHg) and bulbar (81.6 ± 28.6 mmHg) phenotypes. Resting pressure of UES (pUES) was non-significantly correlated negatively with PAS (−0.24) and positively correlated with ALSFRS-R (+0.35) and MRC score (+0.37). No correlation was observed with P-Score (−0.09) (Table 2). In three patients (two bulbar and one spinal) a UES spasm with incomplete sphincter relaxation was detected during manometry (Figure 1A). All of them complained of oropharyngeal dysphagia, confirmed at laryngoscopy FEES (PAS 2.6 ± 1.1, P-Score 5.3 ± 1.15) and had delayed gastric emptying. None of the controls showed a UES spasm. An incomplete UES relaxation (pUES > 5 mmHg) was found in 5 of the 12 ALS patients (Figure 1B) and 2 of the 14 controls (*p* = 0.1).

**Table 2:** Correlation of UES resting pressure with neurological and FEES score of ALS patients and spinal and bulbar onset.

	**pUES (mmHg)**			
	**BULBAR (*****n** =* **5)**	**SPINAL (*****n** =* **7)**	**Pearson**	***P*** **-value**
	81 ± 28.6	58 ± 43		0.3
ALSFRS-R	42.4 ± 4.7	37.7 ± 4.4	+0.35	0.2
MRC (global score)	146.6 ± 5.6	117.5 ± 23.3	+0.37	0.2
PAS	1.6 (±0.5)	2.3 (±1.9)	−0.24	0.4
P-score	2.4 (±3.1)	2.6 (±3.2)	−0.09	0.78

pUES, upper esophageal sphincter resting pressure; ALSFRS-R, Amyotrophic Lateral Sclerosis Functional Rating Scale; MRC, Medical Research Council Scale for Muscle Strength; PAS, Penetration Aspiration Scale; P-score, Pooling score.

Resting LES pressure and complete LES relaxation did not significantly differ between ALS patients with bulbar and spinal onset (15.8 ± 11.9 mmHg vs. 20.6 ± 11.9 mmHg, *p* = 0.5) and controls (14.8 ± 11.2 mmHg, *p* = 0.33). The assessment of esophageal motility during wet swallowing showed a normal manometric pattern in seven patients, ineffective peristalsis in two patients, aperistalsis, diffuse esophageal spasm, and hypercontractile esophagus in one patient each. No significant differences were found between manometric findings and clinical features of ALS nor disease duration (22 ± 12 and 23.6 ± 22.6 months *p* = 0.08) and clinical disability (ALSFRS-R 39.8 ± 5.1 vs. 39.9 ± 5.2 *p* = 0.09; MRC score 130.6 ± 15.8 vs. 127 ± 29.5 *p* = 0.08).

The age at ALS onset was higher in patients with abnormal esophageal manometry (71 ± 10.2 years) compared to those with normal esophageal manometry (54.2 ± 9.5 years), irrespective of ALS phenotypes (*p* = 0.01).

### Gastric emptying measurements

**Figure 3:**
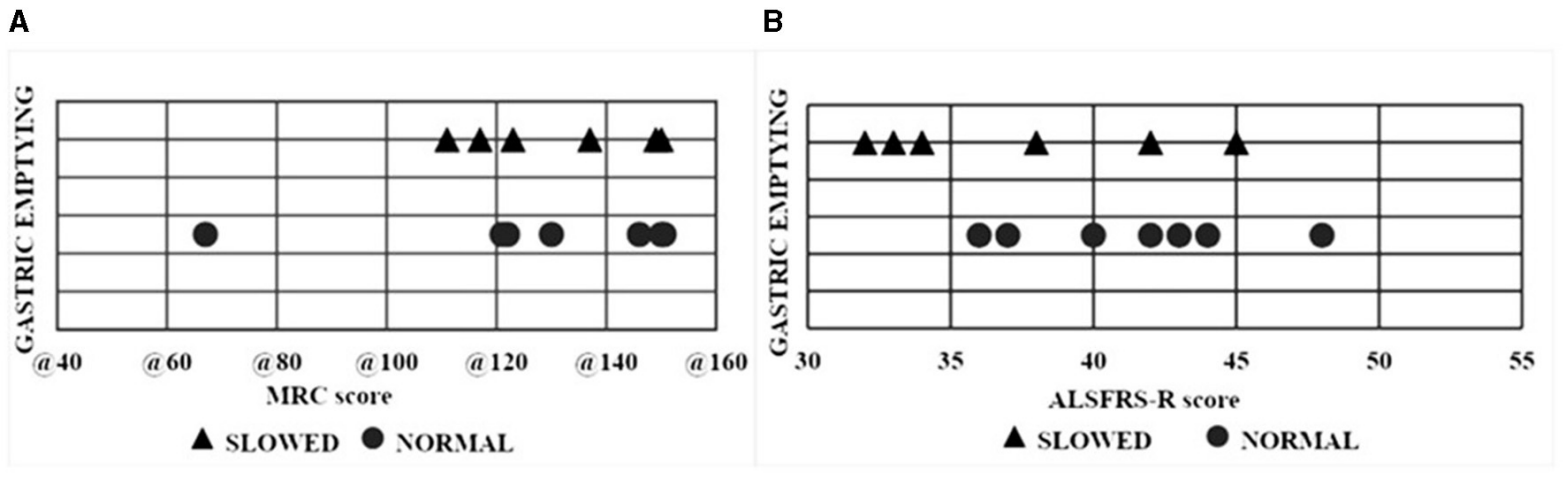
Scatter plot of each ALS patient with normal and delayed gastric emptying. **(A)** Relationship between gastric emptying and MRC. **(B)** Relationship between gastric emptying and ALSFRS-R scores. ALS, Amyotrophic lateral sclerosis; MRC, Medical Research Council Scale for Muscle Strength; ALSFRS-R, ALS Functional Rating Scale.

All 13 patients ate their test meal within 25 min (range: 10–25 min). Delayed gastric emptying (DGE) was found in two patients with bulbar and in four patients with spinal onset. Patients with delayed gastric emptying were more elderly (age 70.3 ± 9 years) compared to those with normal gastric emptying (50 ± 10.47 years) (*p* = 0.012) (Figure 2A). Gastric emptying was not significantly correlated with ALS phenotypes nor with the duration of the disease (Figure 2B). The ALSFRS-R score and the MRC score were not associated with the modality of gastric emptying (Figure 3).

### GI symptoms

At the Rome III questionnaire, three patients with bulbar and three with spinal onset complained of constipation. Two patients with spinal onset reported regurgitation and one heartburn and one dyspeptic symptom. One patient with bulbar onset complained of heartburn and dyspeptic symptoms. There was no association between GI symptoms and DGE. Four of the patients with bulbar onset (80%) and two of the patients with spinal onset (25%) reported dysphagia, FEES detected dysphagia in five patients with no difference between spinal (three patients) and bulbar (two patients) phenotypes. Specifically, four patients had oral dysphagia and one pharyngeal dysphagia (Table 3).

**Table 3:** Upper and lower gastrointestinal symptoms in ALS patients with spinal and bulbar onset.

	**BULBAR (*n =* 5)**	**SPINAL (*n =* 8)**
Heartburn	1^*^	1
Regurgitation	0	2
Dyspepsia	1^*^	1
Dysphagia	2^*§^	3^§°^
Constipation	3^*^	3

No patient complained of diarrhea. ^*^One patient with bulbar onset complained of heartburn, dyspepsia, dysphagia, and constipation. ^§^Four patients had oral dysphagia and one °pharyngeal dysphagia.

## Discussion

Amyotrophic lateral sclerosis (ALS) is a neurodegenerative and multisystemic disease that limits the quality of life and survival of affected patients.

Weight loss, malnutrition, and severe dysphagia are negative prognostic factors for survival. In addition, reduced caloric intake due to dysphagia and increased energy and metabolic requirements (18), weight loss may also be due to the presence of visceral gastrointestinal motor abnormalities.

Several studies showed autonomic involvement in ALS (11). ANS dysfunctions characterized by abnormalities of the cardiovascular system, sweat glands in skin biopsies, and salivary and lacrimal gland nerves have been documented in ALS and are suggestive of ANS degeneration (19–21).

In the present study half of ALS patients have abnormalities of the UES and esophageal peristalsis with normal LES resting pressure values. Hypotonia, incomplete relaxation of the UES, and episodes of spasm are associated with poor prognosis in ALS as factors that can trigger aspiration (8). In our study, we observed an increase in UES resting tone compared to controls, confirming the results of a previous study (22) as well as spasms with incomplete relaxation of the UES in three patients.

MacDougall et al. (23) failed to demonstrate UES spasm on pharyngoesophageal manometry in ALS and found a significant decrease in UES pressure during wet and solid swallowing in 7/13 patients with dysphagia. An explanation for all these different results could be the different ALS patients studied. We included patients with an early- intermediate disease stage and a median disease duration of 10 months. It is plausible that we assessed UES at an earlier stage of ALS disease compared with Higo's study (8).

The early to intermediate stages of ALS, as determined by the ALSFRS-R and disease duration typically exhibit preserved functional capacities, although subtle autonomic and gastrointestinal abnormalities may already be present. Expanding in future studies the patient cohort and incorporate a formal ALS staging framework—encompassing early, intermediate, and late stages—may better capture the dynamic progression of these findings. For instance, the observed increase in UES tone and delayed gastric emptying in some patients may represent early indicators of autonomic involvement, which could evolve as the disease progresses.

Like esophageal achalasia, it is possible to hypothesize a progressive variation of UES disorder in ALS. In esophageal achalasia, it has been suggested that the different manometric patterns represent different stages of the disease (24). According to the “evolutive pattern theory” of the disease, in ALS patients, hypertonia of the UES may represent the initial phase, incomplete relaxation and spasms the intermediate phase, and hypotonia the final phase of the UES disorder. The negative correlation with PAS supports this hypothesis even in the absence of statistical significance due to the small sample size. Hypertonia, as an initial stage of UES involvement, appears to be less frequently associated with aspiration in ALS while UES spasm causes aspiration (8). Three ALS patients with UES spasms and incomplete relaxation reported oro-pharyngeal dysphagia and all had delayed gastric emptying, suggesting an ANS dysfunction. The involvement of the vagus in causing UES spasm is suggested by the observation that UES spasm with severe dysphagia follows bilateral cutting of the vagus in the dog and excision of a cervical vagal schwannoma in humans (25).

However, it should be highlighted that we used conventional perfusion manometry in five patients which does not record faster contractions of striated UES muscle. In addition, Higo et al. (8) used videomanofluorometry to assess UES function, a technique that allows to assess swallowing function more accurately than manometry alone.

Used in less than half of patients, conventional manometry lacks the temporal and spatial resolution of HRM, which is crucial for detecting rapid UES muscle contractions and subtle esophageal motor dysfunctions. This limitation likely led to an underestimation of UES spasms and other subtle abnormalities. In our study UES spasms exclusively in ALS patients and did not in controls. Nevertheless, conventional manometry not differently from HRM identifies significant esophageal motor dysfunction, including ineffective motility and aperistalsis. We particularly found heterogeneous disorders of esophageal motility without significant correlation with dysphagia and clinical features of ALS. In one patient, we found deficient contractility of the esophagus mimicking achalasia, as in a case report (26) of a 53-year-old ALS patient who developed dysphagia due to degeneration of parasympathetic efferent fibers of the DMV.

Papadopoulou et al. (9) recently confirmed the presence of autonomic dysfunction in 21 ALS patients by demonstrating atrophy of the vagus independent of bulbar involvement. Compared to healthy controls, ALS patients exhibited a smaller cross-sectional area of both the right and left vagus as measured by the US at the level of the thyroid gland, where the body of the vagus consists of parasympathetic fibers arising from the dorsal nucleus. Previous studies have described atrophy of the vagus nerve in patients with bulbar-only ALS, attributing the atrophy to the involvement of the nucleus ambiguous. In contrast, the Papadopoulou study suggests that the involvement of the ANS can occur independently of the involvement of the motor neurons (9).

The age at ALS onset was higher in patients with abnormal esophageal manometry (71 ± 10.2 years) compared to those with normal esophageal manometry (54.2 ± 9.5 years), irrespective of ALS phenotype. This finding should refer to the age-related changes in esophageal motor activity (27) more than to the ALS disease. Conversely, UES spasms were likely attributable to ALS, as they were absent in controls and are not commonly described in the elderly population. Age-related UES abnormalities typically involve delayed swallowing response, altered hyoid and laryngeal elevation dynamics, and incomplete UES opening, but not the spasms. To note, the hypertonia or spasm of UES may also result from upper motor neuron (UMN) dysfunction, as it disrupts the normal inhibitory signals to the lower motor neurons. The loss of inhibitory control from UMN dysfunction can exacerbate UES abnormalities, resulting in impaired swallowing mechanics due to increased resistance at the UES contributing to the impaired swallowing mechanics observed in ALS patients (28).

A few previous studies have examined gastrointestinal motor function in ALS.

Toepfer et al. (10) showed delayed gastric emptying, evaluated using a 13C-octanoic acid breath test, in 15/18 ALS patients. In our study, delayed gastric emptying was found in six patients, two with bulbar, and four with spinal phenotype, respectively. Clinical phenotype did not correlate with gastric emptying. Disease duration is significantly greater in ALS patients with spinal compared to bulbar phenotype but, according to the Toepfer study, there was no association between DGE and disease duration. Differently from the Toepfer study we evaluated disability using ALSFRS-R and MRC scores. As expected, the MRC score was lower in patients with spinal compared to bulbar onset whereas there was no difference in ALSFRS score.

Three patients with age at the diagnosis ≥74 years had delayed gastric emptying. This finding should refer to the age-related changes in gastric motor activity more than to the ALS disease (29).

Even if technetium-99-labeled meal scintigraphy remains the gold standard (30) to evaluate gastric emptying, it is an invasive method and difficult to use in patients with neurological diseases. The method requires patients to remain under a gamma camera for extended periods, which can be particularly challenging for ALS patients due to their physical discomfort and difficulty in maintaining a fixed posture. These constraints may not only affect patient compliance but could also compromise the reliability of the results in this population. In contrast, US provides a non-invasive, patient-friendly alternative that is especially suitable for vulnerable populations with neurological diseases such as Parkinson's disease and ALS. The US technique has been validated with scintigraphy and after intragastric administration of known amounts of liquid (17, 30). The technique has been proven to be repeatable and it allows for the assessment of gastric emptying after a physiologically relevant meal and requiring only a few minutes to obtain serial measurements of the antrum.

It is noteworthy that patients with ALS commonly experience GI symptoms. In the present study, the most common GI symptoms were dysphagia (46%) and constipation (46%) of patients, respectively. UES and esophageal motility disorders may contribute to dysphagia, independently from the ALS subtype.

We did not find a significant correlation between gastric emptying, esophageal motor activity, and the ALSFRS-R and MRC scores. The lack of association with the MRC score, assessing upper and lower limb muscle strength, confirms the data suggesting that ANS involvement is independent of motor symptom severity (9). The lack of association between delayed gastric emptying and esophageal motor activity abnormalities and ALSFRS-R raises questions about the role of GI autonomic alteration on the overall function of patients with ALS. In ALS patients, indeed it has been shown a reduction of heart rate variability (HRV) associated with a degeneration of DMV and nucleus ambiguous (NA) (31).

It is important to underline that the laryngeal sensitivity plays a significant role in neurogenic dysphagia (2, 7) raising the question of ANS involvement in motor neuron disease. Decreased laryngeal sensation, though present in a minority of ALS patients, may significantly contribute to the dysphagia observed in this cohort. This sensory deficit can impair the protective reflexes necessary to prevent aspiration during swallowing. Previous studies, such as that of Ruoppolo et al. (2), have shown that laryngeal sensory impairment is associated with dysphagia and aspiration risks in ALS patients. These findings highlight the importance of evaluating both sensory and motor dysfunction in understanding the multifactorial nature of swallowing difficulties in ALS.

Finally, cognitive impairments, particularly those within the spectrum of frontotemporal dementia (FTD), may play a critical role in the clinical manifestations of ALS. Patients with FTD-associated ALS often demonstrate a reduced awareness of dysphagia and aspiration, which may delay symptom reporting and management (32). We did not evaluate the cognitive status of patients, nonetheless the potential contribution of mild cognitive impairments to symptom severity and clinical outcomes cannot be excluded. Including cognitive evaluations in future studies could help clarify the relationship between FTD, dysphagia, and GI motor abnormalities in ALS.

The relatively small number of patients included does not allow us to distinguish pathophysiological differences between bulbar and spinal-onset ALS. However, even if patients with bulbar-onset reported dysphagia more frequently than those with spinal onset, FEES did not show significant differences between patients with different phenotypes. Furthermore, also resting LES pressure, UES and motor findings did not differ in patients with bulbar and spinal onset. Future studies including more numerous patients population are needed to confirm our preliminary observations and to assess phenotype-specific differences.

## Conclusions

The presence of UES spasms and delayed gastric emptying at an early stage of the disease suggests vagus nerve involvement and confirms the hypothesis that ALS should be considered a multisystemic disease. Results of the present study do not confirm previous findings indicating the onset of autonomic symptoms/signs in the late phase of the disease (9, 10), however, the small number of patients evaluated does not allow us to draw definitive conclusions even if it should be noted that the patients included had moderate disease severity. Finally, even though about one-third of the patients reported swallowing difficulties during the visit, all of them consumed a daily caloric solid meal in normal time.

## Data Availability

The raw data supporting the conclusions of this article will be made available by the authors upon reasonable request.
